# Three-Phase Coexistence in Lipid Membranes

**DOI:** 10.1016/j.bpj.2016.12.025

**Published:** 2017-01-24

**Authors:** Anders Aufderhorst-Roberts, Udayan Chandra, Simon D. Connell

**Affiliations:** 1 School of Physics and Astronomy, University of Leeds, Leeds, United Kingdom

## Abstract

Phospholipid ternary systems are useful model systems for understanding lipid-lipid interactions and their influence on biological properties such as cell signaling and protein translocation. Despite extensive studies, there are still open questions relating to membrane phase behavior, particularly relating to a proposed state of three-phase coexistence, due to the difficulty in clearly distinguishing the three phases. We look in and around the region of the phase diagram where three phases are expected and use a combination of different atomic force microscopy (AFM) modes to present the first images of three coexisting lipid phases in biomimetic cell lipid membranes. Domains form through either nucleation or spinodal decomposition dependent upon composition, with some exhibiting both mechanisms in different domains simultaneously. Slow cooling rates are necessary to sufficiently separate mixtures with high proportions of l_o_ and l_*β*_ phase. We probe domain heights and mechanical properties and demonstrate that the gel (l_*β*_) domains have unusually low structural integrity in the three-phase region. This finding supports the hypothesis of a “disordered gel” state that has been proposed from NMR studies of similar systems, where the addition of small amounts of cholesterol was shown to disrupt the regular packing of the l_*β*_ state. We use NMR data from the literature on chain disorder in different mixtures and estimate an expected step height that is in excellent agreement with the AFM data. Alternatively, the disordered solid phase observed here and in the wider literature could be explained by the l_*β*_ phase being out of equilibrium, in a surface kinetically trapped state. This view is supported by the observation of unusual growth of nucleated domains, which we term “tree-ring growth,” reflecting compositional heterogeneity in large disordered l_*β*_ phase domains.

## Introduction

A major advance in membrane science in recent decades has been the realization that multicomponent membranes are not randomly mixed, but are laterally heterogeneous owing to lipid-lipid interactions (1). Although there are still many open questions, it appears likely that these lipid-lipid interactions influence the formation of nanodomains, which in turn function as platforms for membrane protein translocation (2), cell-signaling (3), and receptor desensitization (4). An established way to understand the conditions under which domains form is to compile a phase diagram based on experimental observation, using a model lipid system comprising just three components (5). Such model systems not only give insights into the more complex phenomenon of lipid organization in native membranes but also have their own applications, which include biosensing (6), drug-delivery (7), and nanofabrication (8).

Many studies have been carried out that collectively have helped to build up a ternary phase diagram of such lipids. Phase diagrams of ternary mixtures have been widely explored by a number of different groups (9). Typically, a ternary mixture is selected that comprises cholesterol, an unsaturated lipid such as dioleloylphosphatidylcholine (DOPC) (10) or palmitoyloleoylphosphatidylcholine (POPC) (11), and a saturated lipid such as dipalmitoylphosphatidylcholine (DPPC) (10) or distearoylphosphatidylcholine (DSPC) (12). A number of studies have also used sphingomyelin (13) as the saturated lipid component, which, although somewhat more complex in composition, carries with it the advantage of being a more realistic representation of the outer leaflet of the plasma membrane. Although phase diagrams do vary according to the lipid being studied, in each of these mixtures, the phase behavior is broadly comparable at room temperature (9). For example, if cholesterol content is relatively high, the membrane generally separates into a disordered liquid phase (l_d_) and an ordered liquid phase (l_o_). At low cholesterol content, phase separation instead occurs between l_d_ and an ordered gel phase (l_*β*_) that is enriched in saturated lipids. At intermediate cholesterol concentrations, it has been inferred that a region of three-phase coexistence exists (14). In direct contrast to the extensive body of literature on the l_o_/l_d_ and l_*β*_/l_d_ regions of the phase diagram, there have been almost no studies of the three-phase region.

A number of studies have compiled ternary phase diagrams from three-component lipid mixtures. For example, ternary phase diagrams using sphingomyelin; DOPC, and cholesterol have been calculated using electrofusion of vesicles (15) and by visualization of giant unilamellar vesicles (GUVs) (16). Both studies report a horizontal boundary between the l_*β*_-l_d_ two-phase region and the l_*β*_-l_o_-l_d_ three-phase region, suggesting equal partitioning of cholesterol into the l_*β*_ and l_d_ phases. Similar results have been reported for equivalent ternary mixtures that instead use DPPC (5) and in native fibroblast membranes (17), suggesting that the phenomenon may be universal to gel domains. However, there exist other reports (18) where such boundaries are not observed to be horizontal, and thus, this remains a matter of contention.

Researchers have generally identified the three-phase region by decomposing NMR spectra into three components (18, 19) and have mapped its boundaries by extrapolating from tie lines outside the three-phase region (5). There have, however, been very few reports of successful imaging of three-phase behavior. In one study, three-phase behavior was observed in just a single vesicle (20), whereas in another study, it was reported with the charged lipid dioleloylphosphatidylglycerol (DOPG) rather than DOPC, under specific buffer conditions (21). Thus, there remains a notable lack of understanding of three-phase separation.

One reason for this lack of understanding is that the region is hypothesized to be particularly narrow, such that for a given composition, one of the three phases is likely to be present only in very small quantities. In other instances, two of the three phases can appear similar, and it has been suggested that small l_*β*_ domains can be “buried” within larger l_o_ domains (22). Thus, there are also potential issues with sample preparation, particularly with ensuring that all three phases demix effectively. Finding a technique that can distinguish the three phases simultaneously and unambiguously is challenging. Wide-angle x-ray diffraction, for instance, cannot differentiate l_d_ from l_o_ (23), and it is challenging to differentiate l_*β*_ and l_o_ domains in fluorescence images (12). One approach that has the potential to successfully image three-phase bilayers is atomic force microscopy (AFM). Our group has developed AFM protocols that can be effectively used to distinguish bilayer domains whose height differences are on the subnanometer scale using contact and tapping-mode AFM (24). From this data we have also successfully mapped phase boundaries that are in good agreement with published results from other techniques, further confirmation that phase behavior of lipids in planar membrane systems is equivalent to results from nonplanar systems (25).

In this paper we apply these AFM protocols to supported bilayer systems to explore phase separation in a ternary lipid mixture comprising the naturally derived lipid egg sphingomyelin in combination with DOPC and cholesterol in compositions for which three phase coexistence is expected to occur. Samples are prepared carefully to ensure that bilayer lipids are able to diffuse freely (26) due to the presence of a 0.5–2 nm trapped water layer between the bilayer and the substrate that allows lateral lipid diffusion (27). Temperature control is also implemented to ensure efficient demixing of domains and the maintenance of phase equilibrium. We identify three different phases l_d_, l_*β*_ and l_o_ and characterize them not just morphologically but also mechanically, using a relatively new AFM mode known as quantitative nanomechanical mapping (peak-force QNM). This mode provides very fine and absolute control of force to very low levels in liquids, while also eliminating lateral forces as with standard tapping mode (28) and is able to directly measure mechanical properties such as bilayer deformation and tip-sample adhesion.

## Materials and Methods

### Supported bilayer formation

Lipids and cholesterol were purchased in dry form from Avanti Polar Lipids (Alabaster, AL) and solvated to 5 mM in chloroform. Supported bilayers were formed from these lipids by the vesicle-rupture method (29). Specifically, solvated lipids were mixed in a glass vial to the correct molar proportion, dried under a gentle stream of N_2_, and then placed under vacuum overnight to ensure that no chloroform remained. The mixture was then hydrated using Milli-Q water to a lipid concentration of ∼0.5 mg/mL. The suspension was then tip sonicated for 15 min, extruded using an Avanti mini-extruder at a temperature of 50°C, and centrifuged for 3 min. The thoroughness in ensuring complete resuspension of the lipids is motivated by the fact that compositions in the three-phase region have a low proportion of cholesterol and often a high proportion of sphingomyelin, making the initial hydration difficult and also making the multilamellar vesicles stiffer and more resistant to sonication or extrusion.

After this, 100 *μ*L of solution was pipetted onto a freshly cleaved mica substrate along with 50 *μ*L of a solution of 10 mM MgCl_2_. The sample was then incubated in a humid environment at 50°C for ∼1 h, allowing the vesicles to sediment and rupture on the surface to form a continuous bilayer. The elevated temperature was selected to ensure that all lipids were above their main transition temperature. Hence, deposition occurred when all lipids were in a single continuous phase and the composition on the surface was the same as the composition in the solution (30). The presence of a continuous bilayer is important, as recent work has shown that bilayer diffusion is significantly affected within ∼100 nm of a bilayer defect (31), and sensitive phase separation is likely to be even more affected, which may explain the common sight of phase-separated domains being located around the perimeter of bilayer defects (32).

The bilayer was then rigorously rinsed 10 times with 100 *μ*L warm (50°C) water using a Gilson pipette, with the wash directed parallel to the bilayer surface. This was done to remove any remaining vesicles, either in solution or loosely bound to the surface. For all samples, the hydrated bilayer was then placed on a preheated AFM sample stage and cooled down from 50°C to 25°C at a rate of 1°C/min, unless otherwise stated, to allow phase separation to occur gradually and to ensure equilibrium at 25°C. The temperature was then maintained at 25°C when imaging.

### Atomic force microscopy

AFM experiments were performed using a Bruker (Billerica, MA) Fast-Scan Bio AFM equipped with a temperature control stage. Bilayer samples were imaged using contact mode, tapping mode, and force-volume mode, as well as peak-force tapping mode, a relatively recent innovation, in which the AFM controller modulates the *z*-piezo to perform a rapid force-versus-distance measurement several times at each pixel of the image. The probe is therefore in contact with the sample briefly, eliminating lateral forces. Peak-force tapping allows a number of different mechanical parameters to be calculated from the relationship between the force applied and the separation between the sample and the tip, through an AFM mode known as QNM. For this study, we wished to finely control the force being applied, and therefore, both the peak-force setpoint and the gains were adjusted manually. Based on our previous work, we have found that imaging at ∼200 pN produces good-quality images without affecting the sample morphology (25).

When operating in peak-force tapping mode and peak-force QNM mode, cantilever spring constants were first measured by the thermal noise method, and cantilever sensitivity was measured by engaging the cantilever on a hard, clean surface. The tip radius was measured by using a titanium tip characterization standard. The choice of tip was based on a number of factors. First, the probe must not be too sharp (<10 nm), as such tips have a tendency to damage the bilayer, particularly at high forces (>1 nN). It is also desirable to have a high sensitivity to allow fine control of the applied force and to have a low spring constant to allow optimal imaging at low force. Given that sensitivity and spring constant are generally inversely proportional to one another, a trade-off is required. Based on this trade-off, Bruker MLCT-E (k = 0.1 N/m), NP-B (k = 0.12 N/m), and NP-C (k = 0.24 N/m) probes were used. Multiple (∼5–10) separate areas of each bilayer were imaged to ensure that data were representative.

Fractional areas were calculated using the “bearing analysis” tool in Bruker’s proprietary “Nanoscope Analysis” software, and a minimum of 10 sample images at low forces were used to give an average fractional area for each phase. Sample defects and debris, as seen in areas with heights of >5 nm above or below the bilayer surface, were found to be minimal (<2% of any given image) and were ignored for the purposes of measuring domain areas.

## Results and Discussion

### Identification of phases

**Figure 1:**
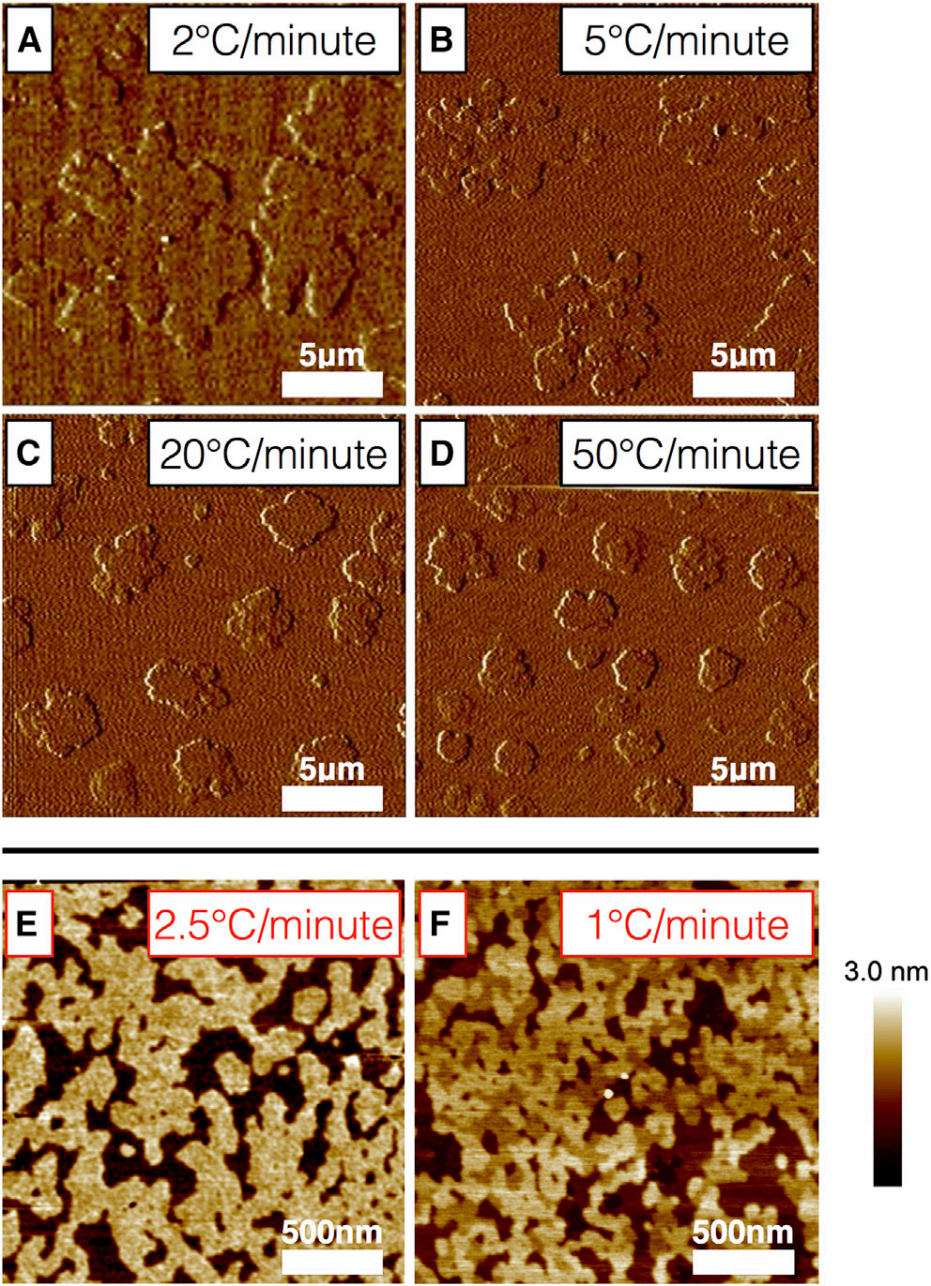
Two-phase and three phase bilayers prepared at variable cooling rates. (*A*–*D*) Contact-mode AFM images (deflection signal) of a lipid bilayer (40% egg sphingomyelin and 60% DOPC) formed at different linear cooling rates, as labeled. Slowing the cooling rate has no effect on height mismatch (1.5 ± 0.1 nm) or domain area fraction (area l_*β*_ = 24 ± 3%), only on the number and size of domains. Cooling more slowly than 1°C/min leads to domains that are too large to observe clearly by AFM at this composition. (*E* and *F*) Three-phase bilayers are imaged with peak-force QNM AFM (composition, 68% egg sphingomyelin, 20% DOPC, and 12% cholesterol). Here, faster cooling causes the bilayer to appear as a two-phase system, although a fine structure is apparent in the higher of the two phases. For slower cooling, the three phases can be clearly seen, suggesting that under increased cooling rates very small domains become kinetically trapped and unable to effectively separate. To see this figure in color, go online.

A number of different compositions within and around the three-phase region were studied using peak-force tapping-mode AFM. Although studying lipid phases in supported lipid bilayers (SLBs) has many advantages, such as the SLB’s ideal geometry for applying a wide variety of scattering and local probe techniques, and the averaging and annealing of a large population of absorbed vesicles, hence negating the variation in composition seen in GUVs (33) it also has some disadvantages. It is only possible to accurately measure phase boundaries and hence plot phase diagrams when the system under study is fully at equilibrium. With liquid phase co-existence (l_o_-l_d_), this is straightforward, as the domains are relatively mobile, even in SLBs. Solid and highly viscous phases such as the l_*β*_ phase, on the other hand, require slow cooling rates with long periods of equilibration. For example, in a study of a mixture of N-palmitoyl sphingomyelin (PSM) and N-palmitoyl ceramide (PCer), both high-transition-temperature lipids that form solid phases at room temperature, with POPC in the form of multilamellar vesicles (34), where three phases were observed, 1 h was required to reach full equilibrium. In another study of phases in GUVs consisting of DOPG (a charged lipid), SM, and cholesterol, an equilibration time of 2 h was required to observe complete separation of the l_o_-and-l_d_ phase into one coalesced l_o_ domain and one l_d_ domain (21). Although the proximity of the substrate decreases the diffusion constant of the individual lipid molecules by only a small degree, it has a much larger effect on domains, introducing a drag effect. This almost halts equilibration of the phase structure once the domains have formed (within a realistic observable time frame). The solution is to use a very slow cooling rate so that the system remains as close to equilibrium as possible during the development of the phase structure. Unfortunately, with l_*β*_ phases, this leads to the formation of sparse and very large domains, too large to be observed by AFM, which is an inherently high-resolution technique. A recent study of the kinetics of solid domain growth in a DOPC/DPPC binary system (35) indicates that in the development of solid-phase domains, a cooling rate of 5°C/min is considered rapid, 1–5°C/min is relatively fast, and 0.1°C/min is very slow and considered ideal. To investigate the effect of cooling rates on SLB bilayer morphology, we performed experiments on a well characterized binary system that phase separates into a solid and a liquid phase (40% egg sphingomyelin and 60% DOPC), similar to the system examined in the study just mentioned (35). The bilayer was prepared in the usual manner, heated in an open AFM cell to 50°C, then cooled at variable controlled cooling rates from 120°C/min down to 1°C/min, as shown in Fig. 1, *A*–*D*. The different linear cooling rates were achieved using an adapted Linkam heat-cool stage, consisting of a silver block sample holder through which liquid nitrogen is pumped, the cooling balanced with resistive heating under feedback control. The same location on the sample is shown in each image between repeated heat-cool cycles. The most notable finding was that the slower the cooling rate, the fewer and larger the domains, as expected according to the theory of nucleation and growth. The observation that slower cooling rates lead to larger domains is consistent with study results showing that slower cooling rates are required to form domains large enough to be observed by fluorescence microscopy (16). Changing the cooling rate was not found to affect the height mismatch (1.5 ± 0.1 nm) or the fractional area of l_*β*_ domains (24 ± 3%). As can be observed from the images, this large-scan-range (100 *μ*m) AFM scanner with temperature control is inherently noisy, necessitating the presentation of deflection-error images. Even so, below 2°C/min the domains are becoming too large to observe with higher–resolution, stable AFMs. In addition, long periods at elevated temperatures increase the risk of water evaporation in the open cell destroying the sample. Hence, in this study, we used a cooling rate of ∼1°C/min (unless otherwise stated), which yielded domains of a suitable size for AFM study. Although faster than ideal, it is relatively slow and controlled, and allows us to observe phases that are close to equilibrium. After cooling, a constant temperature of 25°C was maintained to ensure comparability with other techniques, as lipid bilayers that are close to phase transition temperatures have previously been shown to behave differently from highly curved membrane systems such as liposomes (36). Samples were stable over a period of hours, indicating that no further development or equilibration of the phase structure took place.

**Figure 2:**
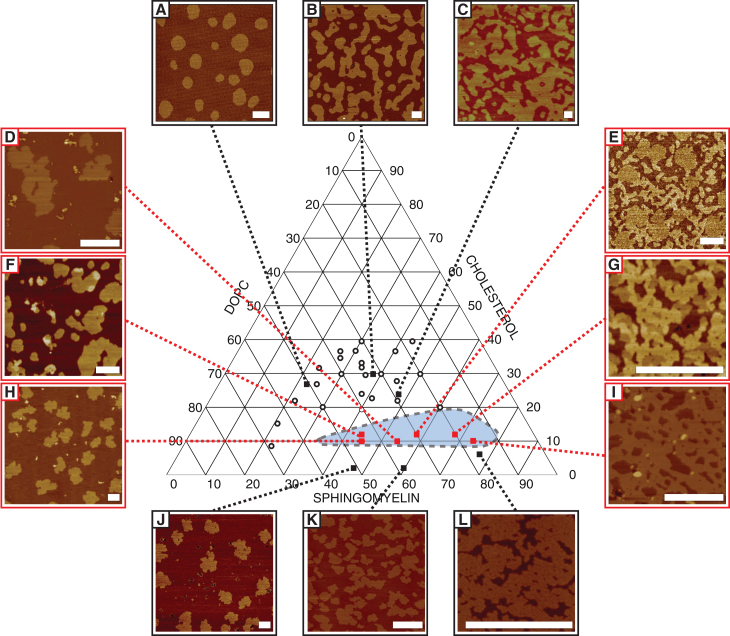
Selected AFM images of bilayers exhibiting two-phase (*A*–*C* and *J*–*L*) and three-phase (*D*–*I*) behavior. Image labels correspond to the compositions listed in Table 1, and black circles are previously published two-phase compositions (25). In the two-phase regions, lateral structure varies gradually and uniformly with composition. By contrast, in the three-phase region (approximated by the *blue shaded region*), lateral structure of the different domains varies significantly between samples, suggesting that nucleation pathways are very sensitive to sample composition. Image sizes are chosen to show fine structure. Scale bars, 1 *μ*m. To see this figure in color, go online.

Three-phase behavior was observed in samples prepared from a range of different lipid compositions. AFM images of a selection of three-phase supported lipid bilayers are shown in Fig. 2. For visual comparison, Fig. 2 also shows a number of images that do not show three-phase behavior. For low cholesterol concentrations (Fig. 2, *J*–*L*), phase separation between l_*β*_ and l_d_ occurs. The l_*β*_ phase is more tightly packed and is thus higher than the l_d_ phase. Due to their ordered packing, the l_*β*_ domains also have rough edges, as their solid nature prevents the flow and reordering of the phase boundary into the lowest-energy configuration, i.e., a circle. At higher cholesterol concentrations (Fig. 2, *A*–*C*), phase separation between l_o_ and l_d_ occurs. Like the l_*β*_ domains, l_o_ domains are also higher than the background l_d_ phase, but their structure appears smoother, because the domains are more fluid. A wider selection of images exhibiting two-phase behavior can be found in our previously published work (25).

At intermediate cholesterol concentrations (∼10–12%), samples exhibit three-phase behavior, and three domains are observed, each with distinct heights. The two highest domains appear to form either binodally or spinodally, depending on composition. For example, for the sample in Fig. 2 *G*, both the highest phase and the second-highest phase appear from their morphology to have formed spinodally, whereas for the sample in Fig. 2 *H*, both appear to be nucleated, suggesting binodal phase separation. One notable case is the sample in Fig. 2 *E*, where both mechanisms are observed: the highest phase appears spinodal and the second-highest phase appears nucleated. We interpret these variations in domain morphology as being an indication that small changes in the sample composition result in significant changes in the trajectory taken through the phase diagram as the sample cools, which in turn results in variable kinetics of domain formation and variable lateral structure.

It could be intuitively assumed that the highest of the three phases is the l_*β*_ phase, as the characteristic close packing of the saturated lipid chains that dominate in the l_*β*_ phase should cause the l_*β*_ domains to stand taller than the surrounding l_o_ and l_d_ phases. However, examination of the area fractions of the three different domains suggests that this may not be the case. The samples in Fig. 2, *D* and *H*, for example, have among the lowest (10%) concentration of cholesterol of any of the samples studied that show three-phase behavior. Thus, their compositions are closest to the l_*β*_-l_d_ two-phase coexistence region, and the bilayer morphologies should show among the highest fractional area of l_*β*_ phase of any of the three phase samples studied. In fact, the fractional areas of the highest phase in the two samples (Fig. 2, *D* and *H*) are measured as 1.11% and 2.81%, respectively. Even without taking into account the variations in area per lipid between the phases, this number is particularly low. Similarly, the sample shown in Fig. 2 *I* has a high concentration of sphingomyelin and thus should have a high l_*β*_ area fraction, with relatively little l_o_. In fact, the intermediate height phase in the sample in Fig. 2 *I* takes up a relatively large proportion of the surface area, whereas the highest and lowest phases take up a relatively low proportion of the surface area. From each of these observations it can be concluded that the highest of three phases is the l_o_ phase, with the second-highest phase being the l_*β*_ phase and the lowest being the disordered (l_d_) phase.

That the l_*β*_ phase appears slightly lower than the l_o_ phase is counterintuitive. In general solid phases are presumed to have a more ordered packing than liquid phases, and thus, they should appear higher, not lower, in AFM images. The consistency of this observation over multiple areas and different modes (contact mode and peak-force tapping, both at low forces) and the striking difference between this and our previously published work on two-phase separation under comparable conditions suggest strongly that this height difference is not an artifact from either poor equilibration or tip-sample interactions.

For samples with high concentrations of sphinomyelin, the three phases appear more difficult to distinguish. The sample in Fig. 2 *I* shows this clearly, with a few high phases buried inside phases of intermediate height and largely isolated from phases of low height. This isolation of one phase from one of the others could result in kinetic trapping of lipids, and thus, samples where this phenomenon is present may be slightly out of equilibrium. This phenomenon was observed to be particularly pronounced when the cooling rate of the bilayer was not controlled.

At quench rates under ambient conditions (no temperature control), measured as ∼2.5°C/min around the lipid transition temperature, T_m_), the mixtures with high proportions of l_o_ and l_*β*_ phases have a very intricate morphology with many small, highly mixed domains, making discrimination between phases difficult, even with high-resolution AFM. This would of course prove equally problematic for other experimental techniques. An example of this phenomenon can be found in Fig. 1, *E* and *F*, which shows a bilayer formed from composition G at a cooling rate of 1°C/min and a bilayer formed from the same composition under a faster, uncontrolled ambient cooling rate. The sample under ambient cooling conditions appears as a two-phase system, although a fine structure can be seen in the higher of the two apparent phases, suggesting that the very small domains are indeed kinetically trapped and unable to effectively separate. When the cooling rate is 1°C/min, the phases separate effectively and can clearly be distinguished. For both samples, the lowest phase occupies a similar fractional area (25.29% for ambient cooling and 26.48% for controlled cooling), suggesting that the sample compositions are identical but that differences in cooling rate cause morphological differences between them.

### Nonequilibrium domain formation

The coexistence of three rather than two phases means that the possibility of phase separated domains becoming kinetically trapped or not fully mixing is increased, due to the possibility of one phase domain becoming buried within a second and in isolation of the third. This is particularly the case if, for example, the kinetics of phase ripening are slower than the kinetics of domain nucleation, such that nano-domains nucleate quickly but the phases coalesce and ripen slowly. To further examine the influence of the kinetics of domain formation on lateral structure, the rate at which the bilayer is cooled after incubation was slowed to 0.4°C/min.

**Figure 3:**
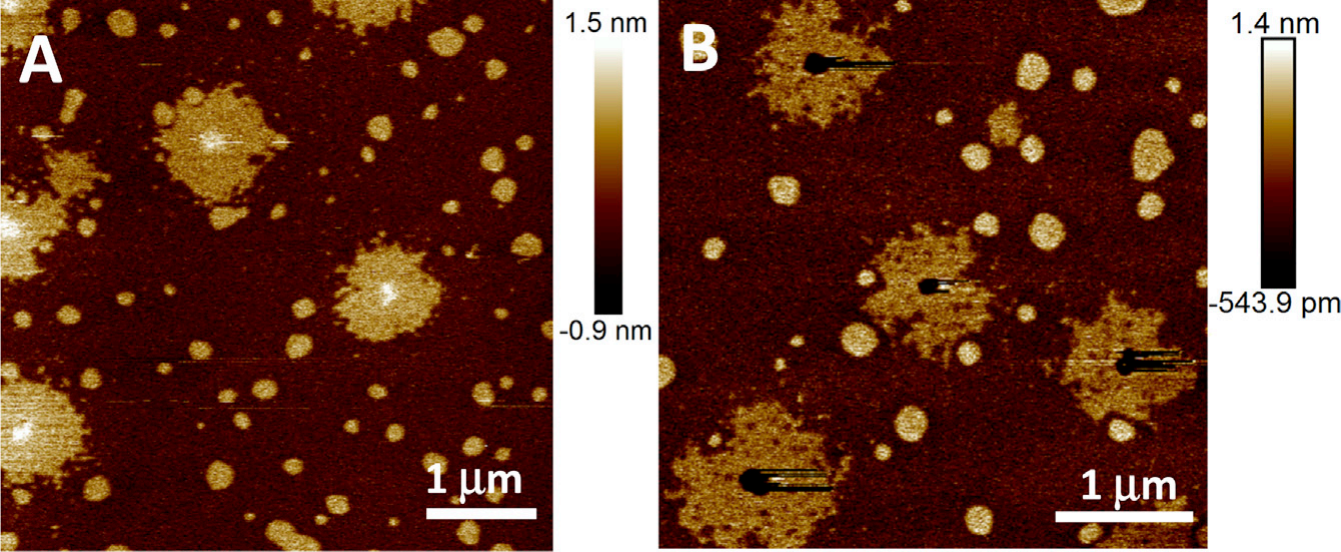
Tapping-mode AFM images of composition F formed at a slower cooling rate of 0.4°C/min. At a high-amplitude setpoint, the core of the l_*β*_ phase appears slightly higher than the surrounding l_*β*_ phase (*A*), whereas at a lower-amplitude setpoint (higher force), the core collapses (*B*), demonstrating variable domain compressibility. Domains are approximately round, suggesting binodal formation. l_o_ domains appear homogeneous, whereas l_*β*_ domains are laterally heterogeneous, suggesting a noncontinuous composition. An image of a larger area of the sample shown in (*B*) can be found in Fig. S1.

Tapping-mode AFM images of the resulting bilayer, prepared from composition F are shown in Fig. 3, *A* and *B*. Fig. 3 *B* shows the bilayer imaged at a “typical” passive setpoint used by our group for other bilayer systems (25). Fig. 3 *A* shows the same sample imaged at a higher-amplitude setpoint, such that force applied by the tip to the bilayer is lower. An image of a larger area of the sample in Fig. 3 *B*, demonstrating a high degree of homogeneity across the sample, can be found in Fig. S1 in the Supporting Material.

The approximately round morphology of the domains in Fig. 3 suggests that they are formed via a nucleation-and-growth mechanism rather than by spinodal decomposition. Furthermore, as seen before, the l_*β*_ phase in Fig. 3 is slightly lower than the l_o_ phase, although the height difference is more subtle at the low setpoint (Fig. 3 *A*), a reflection of the fact that these images are captured using tapping mode, which can lead to difficulty in interpreting small height differences (<1 nm) due to nonlinear complexities in the liquid tip-sample interactions and phase response. The l_o_ domains can also be discriminated from the l_*β*_ domains by their domain morphology. Although both are nucleated, the l_o_ domains are round due to the line tension between phases acting to minimize the domain boundary of the two liquid phases. This tension cannot reorder the solid l_*β*_ phase, which retains its typical solid-phase fractal growth structure. When the AFM setpoint is lowered to a minimum, the core of the l_*β*_ phase appears slightly higher than the surrounding l_*β*_ phase (Fig. 3 *A*), whereas at a higher setpoint, the core collapses (Fig. 3 *B*), thereby demonstrating a clear difference in domain rigidity between the core and the surrounding phase. At higher forces, the rest of the l_*β*_ phase also appears lower than the l_o_ domains, suggesting higher domain deformability.

**Figure 4:**
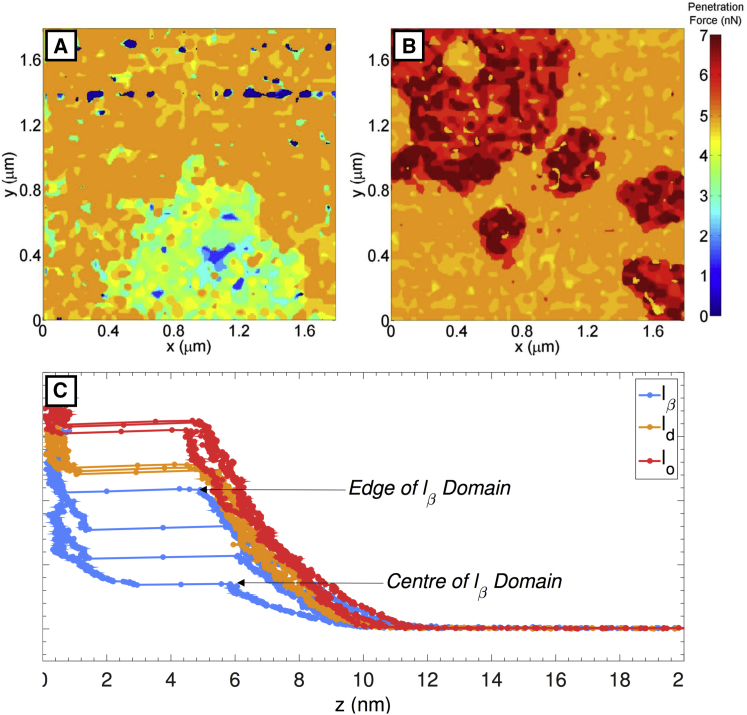
Heat map of the penetration force of the l_*β*_ and l_d_ domains (*A*) and the l_o_ and l_d_ domains (*B*). The l_o_ domains collapse at ∼5.7–6.7 nN (*B*), whereas the l_d_ phase collapses at ∼4.7–5 nN. (*A*) The l_*β*_ domains collapse gradually over a much broader range of forces, 1.4–4.6 nN, with the core of the domains collapsing at low force and the edge at high force; hence, the compressiblity of the l_*β*_ domains varies radially. The penetration force is shown as a discontinuity in the force curves (*C*) and is shown to be approximately constant for different positions in the l_o_ and l_d_ domains but variable across radially different positions in the l_*β*_ domains, further evidence of radial variation in l_*β*_ domain mechanical properties.

To further investigate the l_*β*_ phase’s apparent compositional heterogeneity, the bilayer shown in Fig. 3 was imaged using the force-volume mode, whereby force-distance curves are captured at each image pixel. For each force curve, the force threshold was set to a level at which all phases were observed to collapse, allowing the penetration force to be measured. Fig. 4 shows a heat map representation of the penetration force of the l_*β*_ and l_d_ domains (Fig. 4 *A*) and the l_o_ and l_d_ domains (Fig. 4 *B*). Fig. S2 shows the sequential “slices” from the force-volume-mode AFM at increasing force. Examples of force curves taken at varying positions in the three different domains are shown in Fig. 4 *C*. The apparent softness of the l_*β*_ phase domains necessitates the use of soft cantilevers to prevent the l_*β*_ domains compressing at low forces. However, to induce compression, high forces are required, corresponding to a high cantilever deflection (>1 V) that is outside the linear range of the AFM’s photodetectors. This results in force-distance curves that are nonlinear at the initial point of contact between probe and bilayer. This nonlinearity means that differences in moduli between domains cannot be easily resolved. However, the force at penetration, manifested as a discontinuity in this force curve, can clearly be measured.

Fig. 4 shows that the l_o_ domains collapse at ∼5.7–6.7 nN, whereas the l_d_ phase collapses at ∼4.7–5 nN. These values are both in close agreement with previous work under similar conditions (37). The l_*β*_ domains collapse gradually over a much broader range of forces, 1.4–4.6 nN, with the core of the domains collapsing at the lower part of this range and the edge of the domains collapsing finally at the upper part of this range, at a force similar to that of the l_d_ phase. Therefore, the mechanical properties of the domains can be said to vary radially, which would imply that the domains also have a radially varying composition. It should be noted that the penetration force is not directly related to compressibility. A liquid phase will be compressible due to its fluidity, whereas a solid phase may be initially less compressible, but collapse at a relatively low force. Therefore, the observation that the l_d_ phase is penetrated at a higher force than the l_*β*_ phase is not a contradiction. The force curves in Fig. 4 *C* provide further evidence of radially variable structure, with the penetration force in the l_o_ and l_d_ phases being approximately constant and within the range previously determined, whereas the penetration force in the l_*β*_ phase varies across each domain.

We propose that the origin of this radial variability of l_*β*_ domain composition is caused by a “cored structure” mechanism (38). As the bilayer is cooled gradually, the temperature drops below the phase transition temperature and l_*β*_ domains begin to nucleate. As the temperature drops further, more l_*β*_ phase accretes around this core. The lipids in these l_*β*_ domains are in a solid phase and therefore kinetically trapped; they cannot equilibrate with the overall mixture. The remaining liquid phase is also depleted of the saturated lipids, and hence, phase separation occurs from a different composition. The l_*β*_ domain continues to grow, with each incremental layer having a different composition and therefore different mechanical properties. This process is in many ways analogous to the growth of tree rings. This mechanism is also a commonly observed phenomenon in metallurgy, where it is generally referred to as “coring” (38); however, the process has not as yet been observed in membrane systems.

**Figure 5:**
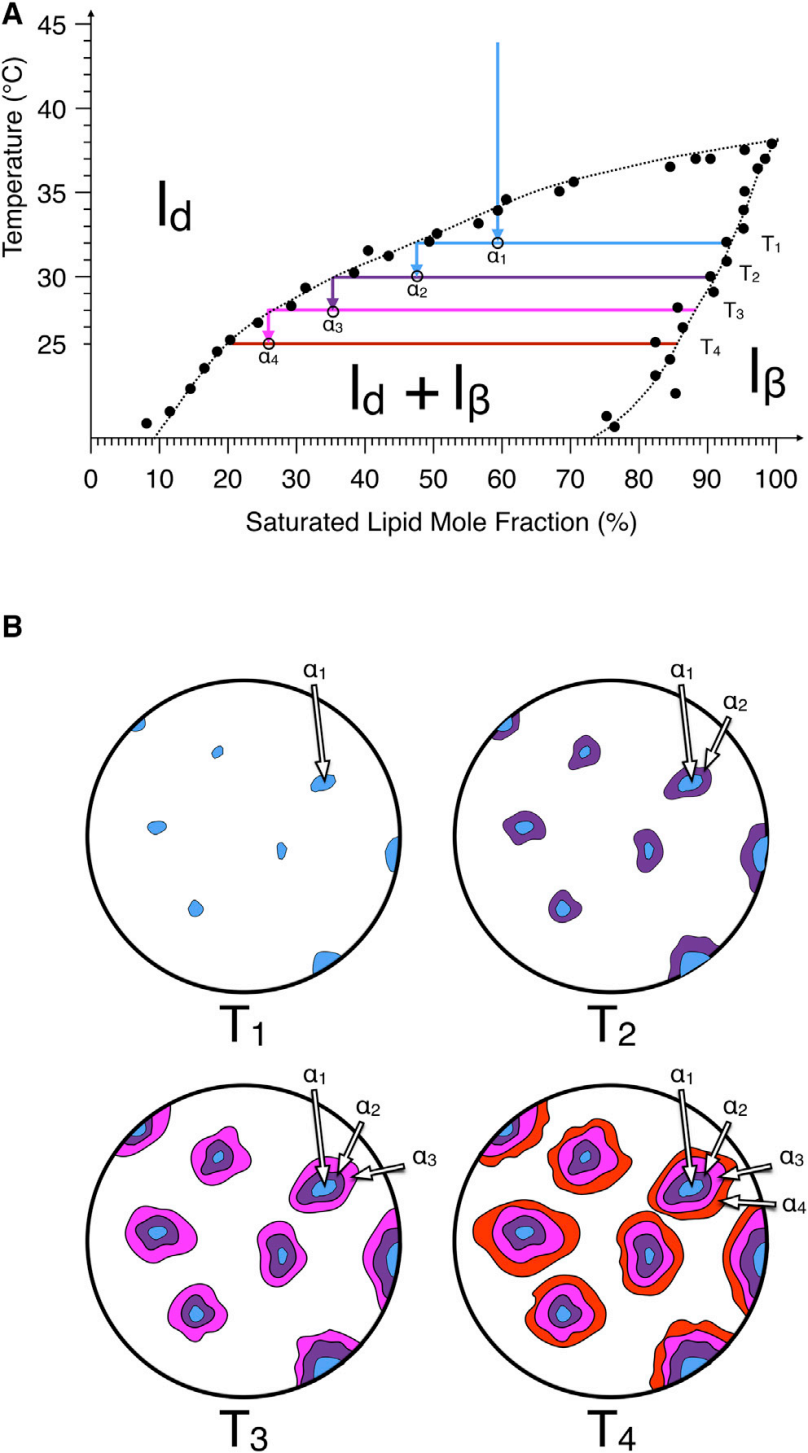
Schematic describing the process of “tree-ring growth.” As a saturated lipid/unsaturated lipid binary-mixture bilayer cools, the temperature drops below the phase transition temperature (*A*; figure for a simple two-phase system as taken from a DOPC/DPPC phase diagram in the literature (39)). With cooling, nucleated l_*β*_ domains begin to appear (*B* (38)). Subsequent drops in temperature result in further phase separation, but only between lipids in the l_d_ phase, as lipids in the l_*β*_ domains are kinetically trapped due to the domain immiscibility. Thus, each incremental layer of the l_*β*_ domain has a subtly different composition and therefore different mechanical properties. Experimentally, the temperature ramp is smooth and continuous; hence, the composition of the domains will follow the solidus curve, and the composition of the melt will follow the liquidus curve.

The “tree-ring growth” mechanism is shown in Fig. 5 in terms of compositional change in the phase diagram and in terms of the resulting lateral heterogeneity. The boundaries of the ternary phase diagram with respect to temperature are poorly understood; hence, a more simplified binary system, in this case DOPC/DPPC (39), is depicted with the general principles of the “tree-ring growth” being the same. This “tree-ring growth” phenomenon was observed for this composition and not for other compositions away from the l_d_ vertex due to the specific characteristics of the phase separation, namely, that the domains are round (nucleated) and large. Thus, these l_*β*_ phase domains have nucleated and then grown from a continuous liquid phase as opposed to forming immediately during spinodal decomposition or nucleating from a background phase that has already itself undergone spinodal decomposition. This nucleation has taken place at a slower rate of 0.4°C/min, allowing it to grow in size. This large size has allowed us to use low lateral resolution force spectroscopy mapping (force volume) to detect differences in penetration force across the single domain, which would be impossible with the smaller domains.

### Mechanical properties of bilayer domains

A more detailed and high-resolution analysis of bilayer mechanical properties can be carried out using peak-force QNM. For composition E, using the standard cooling rate of 1°C/min and peak force of <200 pN, as in Fig. 2, the three phases are distinguished with very clearly nucleated l_*β*_ domains surrounded by spinodally decomposed l_o_ and l_d_ phases (see also Fig. S4). The l_*β*_ domains are measured to be 0.4 ± 0.1 nm above the l_d_ phase (Fig. 7 *A*) and the l_o_ domains are measured as being a further 0.2 ± 0.1 nm above the l_*β*_ domains. These step heights are consistent with the other three phase compositions shown in Fig. 2. A further advantage of using peak force QNM is that the adhesive force between sample and tip and the magnitude of the sample deformation caused by the tip are both directly measured in real time. At the low forces used here both the adhesion between probe and sample and the deformation of the sample are negligible, suggesting minimal tip-sample interaction and minimal sample deformation (see Fig. S3). This is an important finding as it further confirms that the lower than expected height of l_*β*_ is not due to compression caused by tip-surface interactions but rather is an inherent feature of three phase compositions.

**Figure 6:**
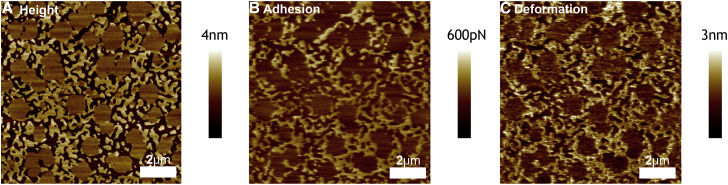
Peak-force QNM AFM image at high force (5 nN) of a phospholipid bilayer formed from composition E. The step heights between the three phases are highly pronounced, indicating that the different domains are compressed to different extents (*A*). The adhesion channel shows high adhesion in the l_d_ phase but negligible adhesion in the other two phases, suggesting substantial compression of the l_d_ domains (*B*). The deformation channel shows that the l_d_ phase is the most deformable, followed by the l_*β*_ phase, whereas the l_o_ phase shows negligible deformability (*C*).

**Figure 7:**
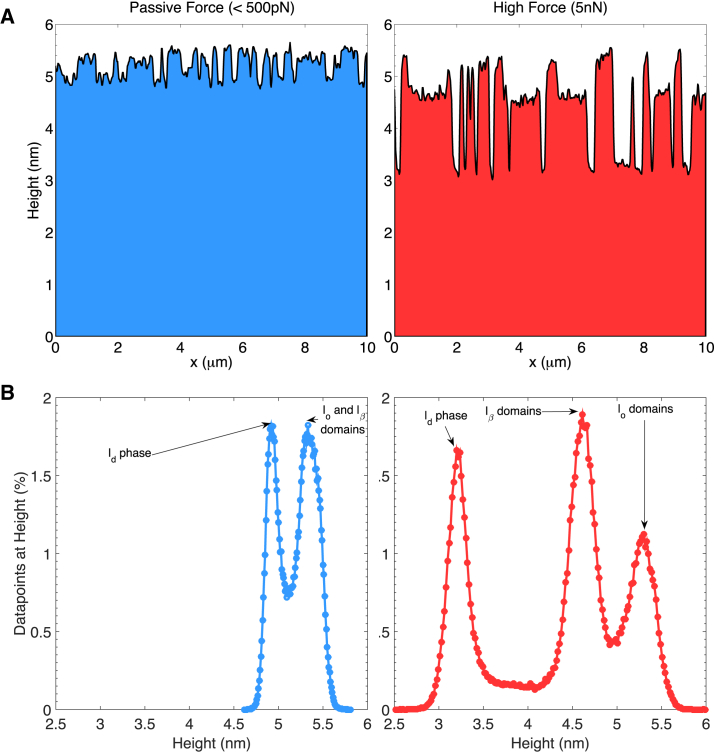
Representative height cross section and image-averaged height histogram of bilayers prepared using composition E at two different forces. (*A*) The step-height differences between phases is shown to increase when the force is increased from ∼200 pN (*left*) to 5 nN (*right*). (*B*) Similarly, at lower forces (*left*), the height histogram shows two distinct peaks, as the l_o_ and l_*β*_ phases are indistinguishable, whereas at higher forces (*right*), differential domain compression results in three clearly distinguishable peaks.

Fig. 6, *A*–*C*, shows the measured height, adhesion, and deformation, respectively, of the same membrane at a higher force of 5 nN. The height image shows a particularly high contrast between the three domains, with large step sizes between domains (see Fig. 7 *A*). The l_*β*_ domains are now 1.4 ± 0.1 nm above the l_d_ phase, and the l_o_ domains are measured as being a further 0.7 ± 0.1 nm above the l_*β*_ domains. Thus, the l_*β*_ domains and the l_d_ phase appear to be compressed significantly by the increased force. The deformation can also be seen laterally, with the l_*β*_ domains in particular observed to increase in area when the force is increased. For this reason, in this study, high forces are not used when calculating the areas of different phases, because the compressibility of the l_*β*_ phase at high forces means that its area is artificially increased. The adhesion image in Fig. 6 *B* shows high adhesion (∼200 pN) in the l_d_ phase only. We postulate that the high peak force causes the cantilever to significantly compress and deform the l_d_ phase, resulting in an increase of the tip-sample contact area, which in turn increases the adhesive force between tip and sample. In the deformation channel, shown in Fig. 6 *C*, the l_d_ phase is measured to be deformed by 2.1 ± 0.2 nm. Similarly, the l_*β*_ domains are measured as being deformed by 1.2 ± 0.2 nm. The relatively high deformability and high adhesion of the l_d_ phase indicates that the l_d_ phase is more compressible than the l_*β*_ domains. The finding from the force spectroscopy data that the l_d_ phase penetrates at higher force than the l_*β*_ domains (Fig. 3), but at the same time is more compressible, is not incompatible. It implies that the membrane failure mechanism is different from the compressive modulus. A liquid phase will deform with a characteristic elastic area compressibility modulus and a zero shear modulus. It can therefore be highly compressible and can resist penetration. The high compressibility leads to a higher tip-sample contact area and hence to increasing adhesion upon retraction. By contrast, a solid phase is characterized by an elastic shear modulus. This can result in its appearing initially stiff, but then being penetrated (sheared) at relatively low force.

The changes in the step height between the domains at different forces are shown graphically in Fig. 7. Bilayer heights are measured with respect to the substrate surface by measuring the depths of defects in the bilayer. Across a number of such defects from different samples, the step height between the substrate and the lowest phase, the l_d_ phase, is measured as 4.9 ± 0.2 nm at passive forces, defined as forces at which sample adhesion and deformation are negligible, and as 3.5 ± 0.2 nm at the higher force of 5 nN. Fig. 7 *A* shows a typical height cross section of the sample at 200 pN (*left*) and 5 nN (*right*), with the three phases being clearly defined at 5nN, but almost indistinguishable at <500 pN. The bilayers were not observed to deform significantly for forces <200 pN.

Similarly, the distribution of heights at the two forces (Fig. 7 *B*) shows that at 200 pN, there are two histogram peaks. The first of these corresponds to the l_d_ phase and the second corresponds to the l_o_ and l_*β*_ phases, whose heights are of similar magnitude such that they cannot be clearly distinguished in the histogram. At a force of 5 nN, the differential compression of the three phases means that all three phases have clearly distinguishable histogram peaks.

Induced deformation of domains appears to indicate a differential in deformability between the three phases. The l_o_ phase is the least deformable, followed by the l_*β*_ phase and finally the l_d_ phase.

### The disordered gel state

Findings in the previous sections have shown that the l_*β*_ phase is less high than the l_o_ phase and also more compressible. This finding is consistent across all data observed in the three-phase region but is somewhat counterintuitive as it is generally accepted that the l_o_ phase is more fluid than the l_*β*_ phase and therefore should intuitively be more deformable (40). The unusually high compressibility of the l_*β*_ domains suggests that this phenonemon is not caused by a systematic tilt of lipids but rather by some reduction in lipid packing order. For comparison, similar phenomena have been reported previously in the literature. In one such example, NMR studies of DPPC-cholesterol membranes showed that increasing the cholesterol concentration of the bilayer caused a sharp component in the ^13^C spectrum, interpreted as being caused by an l_*β*_-like dipalmitoylphosphatidylethanolamine (DPPE) phase whose packing density is disrupted by an increase in cholesterol content (41). A “disordered solid” state has also been reported from wide-angle x-ray-scattered measurements of cholesterol-lecithin bilayers (42) and from a combined NMR and x-ray diffraction study of PC-cholesterol mixtures (12). NMR studies of ternary mixtures have shown that the value of quadropolar splitting in the l_*β*_ state is significantly higher at higher cholesterol concentrations, with the implication being that the excess interdigitation of cholesterol in the ordered l_*β*_ phases causes the hexagonal chain packing to be disrupted, thus leading to a disordered gel state (43). This “disordered gel” state predicts reduced height and reduced packing in sphingomyelin, in line with the results here that show lower l_*β*_ domains with increased deformability, a strong indication that the same phenomenon is responsible.

These NMR data from the literature can be analyzed to give the step height between the observed disordered gel state and the l_o_ and l_d_ states. The sphingomyelin used both in this work and in the NMR study (Avanti egg sphingomyelin, 86%, 16:0) can best be approximated as having an all-*trans* C16 chain, which has a fully extended height of 19.0 Å (43). Further data from the literature indicate that the chain height is reduced to 13.8 Å in the l_d_ state (44), 16.4 Å in the liquid ordered state (43), and 15.3 Å in the disordered gel state (43).

The heagroup height is estimated as being that of a DPPC headgroup (6.7 Å (45)). Thus, assuming that the l_*β*_ domains correspond to the disordered gel state, the full bilayer thicknesses are 4.6, 4.4, and 4.1 nm for the l_o_, l_*β*_, and l_d_ phases, respectively. Assuming that these numbers are accurate, the previously measured height of the l_d_ phase in relation to the substrate, 4.9 ± 0.2 nm, suggests a hydration layer between substrate and bilayer of 0.8 ± 0.2 nm, which is well within the range measured for bilayer systems in the literature (27).

Taking these expected full bilayer thicknesses, the expected step heights between domains are therefore 0.3 nm between the l_d_ and l_*β*_ phases and 0.2 nm between the l_*β*_ and l_o_ phases. When compared to experimentally determined step heights of 0.4 ± 0.1 and 0.2 ± 0.1 nm, respectively, the results from NMR and AFM can be said to be in good agreement.

A second interpretation of these data, and of the rest of the literature described in this section, is that l_*β*_ phases that contain cholesterol are difficult to equilibrate sufficiently with a varying balance of lipids and cholesterol trapped in a nonequilibrium state. This idea is supported by the observation of “tree-ring growth” in certain nucleated compositions (Fig. 3). At high cholesterol concentrations, all phases are liquid and so are able to flow and equilibrate rapidly. With no cholesterol in a binary lipid mixture, the l_*β*_ phase is very ordered, even crystalline in nature, and thus, likewise, no cholesterol is available to become trapped in a nonequilibrium state. This would appear to be a factor with compositions that have both cholesterol and solid phases present. However, this idea does not negate the fact that three coexisting phases are clearly observed in a region of the phase diagram where they were predicted to be. Surrounding mixtures that also exhibit solid phases outside of the three-phase region *never* result in three visible phases, or even surface roughness suggestive of nanometer separation, down to the cooling rates of 0.1°C/min (Fig. 1). This supports the idea that, although perhaps not fully equilibrated, they are real distinct phases. With cholesterol present, the ordering of the l_*β*_ phase is disrupted, leading to softer and lower l_*β*_ domains with a low shear modulus and heterogeneous composition. It is likely that these domains are not quite at equilibrium, and so it is not possible to estimate phase boundaries from the area fraction of each phase.

In addition to the step height, the composition-dependent area per molecule of the different lipid phases based on collated data from the literature can also be calculated using our previously published methodology (25). Here, we define area per lipid for a given phase as the average surface area of lipids in that phase, assuming linear expansion upon heating and taking into account the condensing effect of cholesterol. This approach gives an area per lipid in the three-phase region of 43 Å^2^ for the l_o_ phase. Using the same methodology, the area per lipid of an ordered gel phase is calculated as 39 Å^2^. For the l_d_ phase, there have been a number of recent advances using x-ray scattering, neutron scattering, and modeling that have given a more accurate estimate of the area per lipid as 67 Å^2^ (46). As this figure supercedes our published methodology, we use it as the area per lipid of the l_d_ phase. The area per lipid in the “disordered gel” l_*β*_ domains is calculated by taking the value of 39 Å^2^ for the “ordered gel” state and assuming that the gel state must have a constant volume per lipid. Thus, given that the height of the phase is lower, the area per lipid must increase proportionately. Using NMR data from the literature on chain disorder (43), this gives an area per lipid of 47.9 Å^2^ for the disordered gel state. It is notable that this figure is higher than that for the l_o_ phase but lower than that of the l_d_ phase, reflecting the nature of the disordered gel phase to be less ordered than the l_o_ phase but more ordered than the l_d_ phase.

## Conclusions

Through a range of different AFM modes, we have presented the first images of domains in the three-phase region for ternary phospholipid mixtures containing a saturated lipid, an unsaturated lipid, and cholesterol. High-resolution imaging allows three distinct phases to be clearly discriminated, with structure formation being highly dependent on composition, kinetics, and nucleation pathway. Within the narrow three-phase region, domains undergo phase separation through both spinodal decomposition and nucleation. In some notable cases, both mechanisms are apparent, with nucleated l_*β*_ domains surrounded by spinodal l_o_ domains.

Our results are shown to be consistent with the “disordered gel” state theory, which has long been speculated upon in the literature (43) but is directly observed here. As predicted by prior NMR studies, the increased cholesterol content of the l_*β*_ phase disrupts the hexagonal chain packing of the saturated lipids. Our analysis of these NMR data gives predicted domain step heights that are in excellent agreement with the step height we measure directly using AFM.

Under carefully controlled conditions, radially heterogeneous domains are shown to form, their structure being formed by a proposed “tree ring” model of nucleation and growth. Although this form of growth is commonly reported in the metallurgical literature (38), this study is the first, to our knowledge, to show the phenomenon in membrane systems. Alternatively, the disordered gel phase observed here and in the wider literature could be explained as an out-of-equilibrium phase, where the samples either have not been given sufficient time for phase separation to develop fully or are surface supported bilayers in which the phases are kinetically trapped in a nonequilibrium state. However, this does not negate our main finding that three-phase coexistence has clearly been observed, and that the l_*β*_ phase structure is significantly disrupted by a small quantity of cholesterol.

The aim of this study was to fully characterize the little-studied and elusive three-phase region. The results reveal an incredibly rich phase behavior, where multiple phases may coexist, developing via nucleation and growth or spinodal decomposition mechanisms, or even by both at the same time. Cooling rates affect the development of the structure, and this also depends upon the lipid-substrate interaction, where the surface must hinder domain dynamics to a high degree. Many questions are posed by this study, including the role of phase ripening and the degree of equilibration, and hence the exact location of the phase boundaries with respect to composition and temperature.

## Author Contributions

S.D.C. initiated research; S.D.C. and A.A.-R. designed experiments; A.A.-R., S.D.C., and U.C. performed research; A.A.-R contributed analytic tools; and A.A.-R. and S.D.C. analysed the data and wrote the article.

## Figures and Tables

**Table 1:** Lipid Composition of Each of the Samples in Figure 2

Composition	% DOPC	% SM	% Chol.	3-Phase?
A	51	22	27	No
B	38	32	30	No
C	28	48	24	No
D	36	54	10	Yes
E	30	58	12	Yes
F	44	44	12	Yes
G	20	68	12	Yes
H	45	45	10	Yes
I	18	72	10	Yes
J	52	44	4	No
K	38	58	4	No
L	18	74	8	No

## Supporting Material

Document S1. Figs. S1–S4

Document S2. Article plus Supporting Material
