# Supplementary material for: Three-Phase Coexistence in Lipid Membranes
Source: Biophys J. 2017 Jan 24;112(2):313–24. doi: 10.1016/j.bpj.2016.12.025 (PMC5266263; doi:10.1016/j.bpj.2016.12.025)
Supplement: Document S1. Figs. S1–S4 [file mmc1.pdf]

Biophysical Journal, Volume *112*

Supplemental Information

Three-Phase Coexistence in Lipid Membranes

Anders Aufderhorst-Roberts, Udayan Chandra, and Simon D. Connell

Supplementary Information

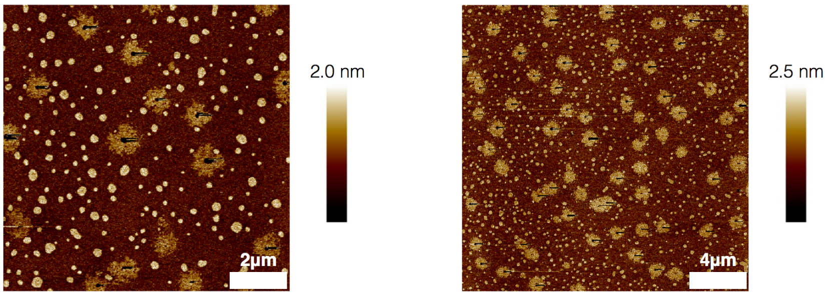

Figure S1: Contact mode AFM image of composition (F) formed at a slower cooling rate of 0.4°C/min at two different scan sizes. Domains are approximately round and evenly distributed suggesting cooling through the binodal with domain formation via a a nucleation and growth mechanism. l_o domains appear homogeneous while l_β domains appear to collapse in their centre demonstrating variable domain compressibility caused by domains being out of equilibrium.

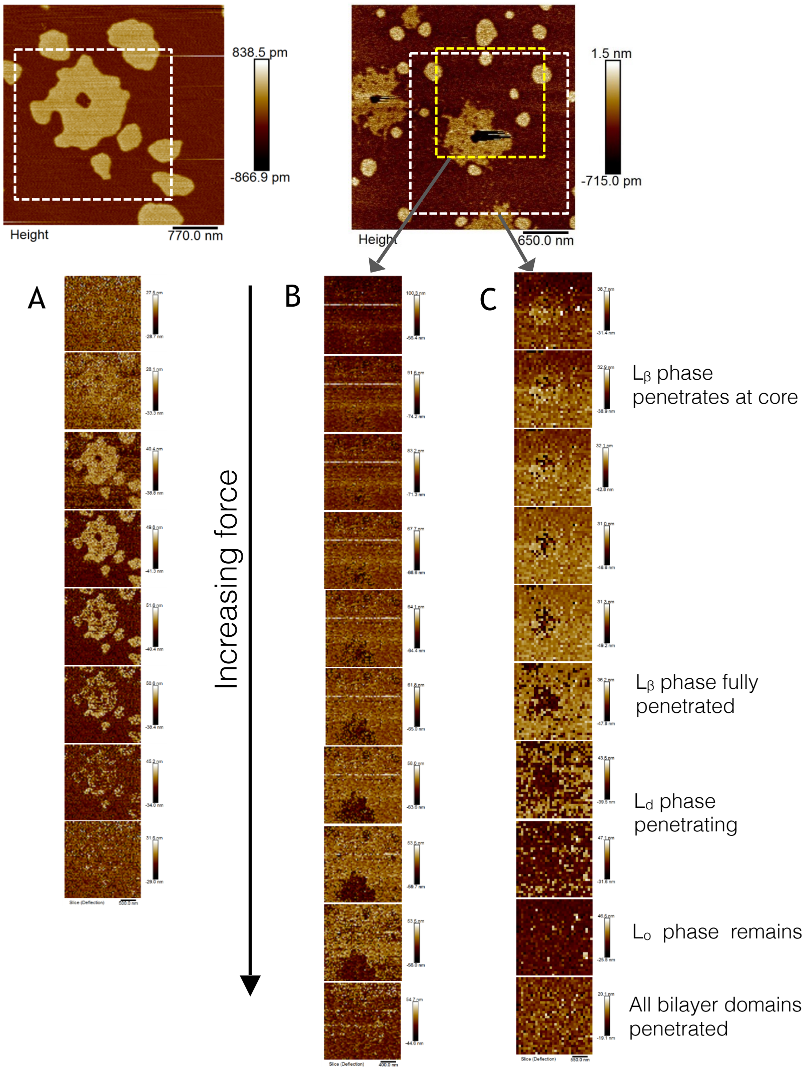

Figure S2: Sequential ‘slices’ at increasing force through force volume mode AFM of three phase images as shown in the heat maps of figure 3, together with standard resolution images of the same area. Sequence A shows large l_o domains only. Three coexisting phases are shown in B and C. In sequence B the maximum force was not set high enough to penetrate the l_o phase. Maximum applied force was increased in the lower resolution Sequence C to capture the l_o penetration event.

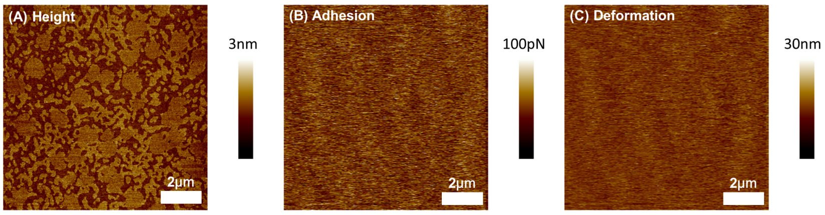

Figure S3: Peak force QNM atomic force micrograph showing the height profile of a phospholipid bilayer formed from composition E. Three phases can be seen, a background l_d phase, a spinodal l_o phase and a circular l_β phase. Both adhesion (B) and deformation (C) as measured by QNM are found to be negligible.

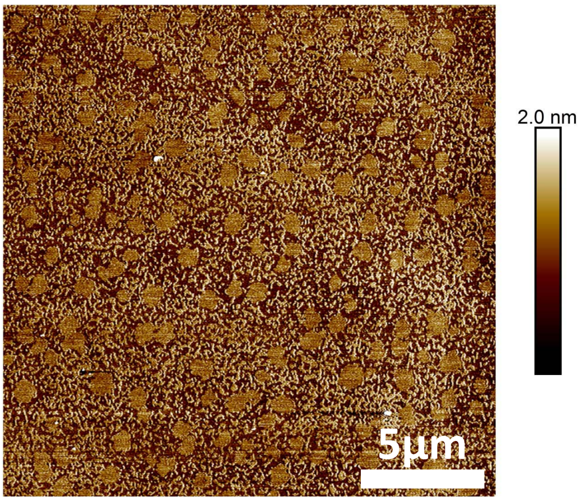

Figure S4: Peak force QNM atomic force micrograph showing the height profile of a phospholipid bilayer formed from composition E over a larger area: 20μm
